# Circulating Tumour DNA Sequencing Identifies a Genetic Resistance-Gap in Colorectal Cancers with Acquired Resistance to EGFR-Antibodies and Chemotherapy

**DOI:** 10.3390/cancers12123736

**Published:** 2020-12-11

**Authors:** Franciele H. Knebel, Louise J. Barber, Alice Newey, Dimitrios Kleftogiannis, Andrew Woolston, Beatrice Griffiths, Kerry Fenwick, Fabiana Bettoni, Maurício Fernando Silva Almeida Ribeiro, Leonardo da Fonseca, Frederico Costa, Fernanda Cunha Capareli, Paulo M. Hoff, Jorge Sabbaga, Anamaria A. Camargo, Marco Gerlinger

**Affiliations:** 1 Translational Oncogenomics Lab, The Institute of Cancer Research, 237 Fulham Road, London SW3 6JB, UK; franciele.knebel@icr.ac.uk (F.H.K.); louise.barber01@icr.ac.uk (L.J.B.); alice.newey@icr.ac.uk (A.N.); andrew.woolston@icr.ac.uk (A.W.); beatrice.griffiths@icr.ac.uk (B.G.); 2 Sociedade Beneficiente de Senhoras Hospital Sírio Libanês, SBSHSL, Rua Dona Adma Jafet 91, São Paulo 01308-050, SP, Brazil; fbettoni@mochsl.org.br (F.B.); mauricio.fsaribeiro@hsl.org.br (M.F.S.A.R.); l.fonseca@fm.usp.br (L.d.F.); frederico.costa@hsl.org.br (F.C.); fernanda.capareli@hsl.org.br (F.C.C.); jsabbaga@uol.com.br (J.S.); aacamargo@mochsl.org.br (A.A.C.); 3 Centre for Evolution and Cancer, The Institute of Cancer Research, 237 Fulham Road, London SW3 6JB, UK; dimitrios.kleftogiannis@icr.ac.uk; 4 Tumour Profiling Unit, The Institute of Cancer Research, 237 Fulham Road, London SW3 6JB, UK; kerry.fenwick@icr.ac.uk; 5 Instituto D’Or de Pesquisa e Ensino, IDOR, Oncologia D’Or, Avenida República do Líbano 611, São Paulo 04.502-001, SP, Brazil; paulo.hoff@oncologiador.com.br; 6 GI Cancer Unit, The Royal Marsden Hospital, 203 Fulham Road, London SW3 6JJ, UK

**Keywords:** colorectal cancer, ctDNA-Sequencing, ctDNA-ddPCR, acquired resistance, genetic resistance-gap, EGFR-antibodies

## Abstract

**Simple Summary:**

Recent studies have shown the potential of next generation sequencing (NGS) for the identification of genetic variants in tumour DNA that has been released into the bloodstream (ctDNA). However, such variants are often rare in the sample and error correction is required to filter out false calls. In this study we used error corrected ctDNA-sequencing to identify genetic drivers of resistance in metastatic colorectal cancer (mCRC) patients that had become resistant to combined chemotherapy and EGFR-antibody treatment. Our data showed that ctDNA-seq could detect common and novel resistance mechanisms in cases of both primary and acquired resistance. ctDNA-seq could therefore facilitate patient stratification to novel therapies and avoid ineffective treatment with EGFR-antibodies. Furthermore, the data revealed a lack of detectable genetic resistance in a large fraction of the cancer cell population, indicating a need to investigate other, potentially non-genetic, resistance mechanisms.

**Abstract:**

Epidermal growth factor receptor antibodies (EGFR-Abs) confer a survival benefit in patients with *RAS* wild-type metastatic colorectal cancer (mCRC), but resistance invariably occurs. Previous data showed that only a minority of cancer cells harboured known genetic resistance drivers when clinical resistance to single-agent EGFR-Abs had evolved, supporting the activity of non-genetic resistance mechanisms. Here, we used error-corrected ctDNA-sequencing (ctDNA-Seq) of 40 cancer genes to identify drivers of resistance and whether a genetic resistance-gap (a lack of detectable genetic resistance mechanisms in a large fraction of the cancer cell population) also occurs in *RAS* wild-type mCRCs treated with a combination of EGFR-Abs and chemotherapy. We detected one *MAP2K1/MEK1* mutation and one *ERBB2* amplification in 2/3 patients with primary resistance and *KRAS, NRAS, MAP2K1/MEK1* mutations and *ERBB2* aberrations in 6/7 patients with acquired resistance. In vitro testing identified MAP2K1/MEK1 P124S as a novel driver of EGFR-Ab resistance. Mutation subclonality analyses confirmed a genetic resistance-gap in mCRCs treated with EGFR-Abs and chemotherapy, with only 13.42% of cancer cells harboring identifiable resistance drivers. Our results support the utility of ctDNA-Seq to guide treatment allocation for patients with resistance and the importance of investigating further non-canonical EGFR-Ab resistance mechanisms, such as microenvironmentally-mediated resistance. The detection of *MAP2K1* mutations could inform trials of MEK-inhibitors in these tumours.

## 1. Introduction

*KRAS* and *NRAS* mutations are predictors of primary resistance to the EGFR-antibodies (EGFR-Abs) cetuximab and panitumumab in metastatic colorectal cancer (mCRC) [1,2,3,4,5]. Furthermore, *RAS* mutations evolve in most mCRCs when they acquire resistance to EGFR-Abs [6,7,8]. Other genetic aberrations that re-activate the RAS/RAF pathway, such as *EGFR* and *BRAF* mutations or *ERBB2* amplification, also confer primary and acquired resistance but are less common [9,10]. Analysing the mutation status of these driver genes in the circulating tumour DNA (ctDNA) through so-called ‘liquid biopsies’ can avoid the need for tumour re-biopsies associated with discomfort, a risk of complications, and high costs. Furthermore, early detection of evolving resistance drivers may help monitor patients and guide personalized treatment switching to alternative therapies. 

Application of liquid biopsies in mCRC patient’s management is becoming increasingly feasible by developing ctDNA-sequencing (ctDNA-Seq) technologies incorporating error correction [11,12], which enable mutation detection in entire gene panels with high sensitivity and low false-positive rates. We developed a ctDNA-Seq assay for CRC patients that applies molecular barcodes (MBC) and duplex DNA identification for error correction and can be performed from 25 ng of ctDNA. We showed that this could call mutations with variant allele frequencies (VAFs) of 0.15% in ctDNA [12]. 

Application of ctDNA-Seq to *RAS* wt mCRC patients who acquired resistance to single-agent cetuximab in the third line setting showed the ability to identify mutations and DNA amplifications that drive resistance [9]. Leveraging the ability of this ctDNA-Seq technique to reconstruct genome wide copy number profiles [12], we assessed the clonality of resistance driver mutations by first correcting VAFs for the influence of copy-number states and by subsequently calculating the proportion of cancer cells that harbored resistance driver mutations by comparing against *TP53* or *APC* mutations, which are likely clonal. This subclonality analysis revealed that only a minority (36%) of cancer cells represented in the ctDNA harbour resistance driver mutations despite radiological progression. This defined a previously undiscovered genetic resistance-gap at the time of acquired cetuximab resistance and led to discovering a novel non-genetic mechanism of single-agent cetuximab resistance, driven by an increase in tumour-associated fibroblasts [9]. 

In this study, we first aimed to validate this ctDNA-Seq technology’s ability using a targeted 40 gene panel to identify mutations in 10 patients who initially showed *RAS* wild-type status in tumour tissue and either showed primary or acquired resistance when treated with EGFR-Ab therapy predominantly in combination with chemotherapy. Moreover, as most patients in this study received a combination of EGFR-Abs and chemotherapy, we investigated what proportion of the cancer cells harboured these drivers to assess if a genetic resistance-gap also occurs in mCRCs that acquired resistance to chemotherapy and EGFR-Ab or if this only arises with single-agent cetuximab.

## 2. Results

Plasma samples were collected after radiologically confirmed progression from ten patients with mCRCs that were *RAS* wild-type based on clinical testing (Table S1). Nine of them had received an EGFR-Ab (cetuximab or panitumumab) in combination with chemotherapy and one single-agent EGFR-Ab (panitumumab) (Table 1). Two (patients 3 and 4) were analysed at the time they were re-challenged with EGFR-Ab therapy. Analogous to previous work [9], we classified patients with progressive disease (PD) within 12 weeks of EGFR-Ab initiation (*n* = 3, the median time to progression: 9 weeks) as cases with primary resistance. Those who obtained a benefit for at least 12 weeks (*n* = 7, the median time to progression: 26 weeks) were considered cases with acquired resistance before they progressed. 

### 2.1. ctDNA Sequencing Results

**Figure 1:**
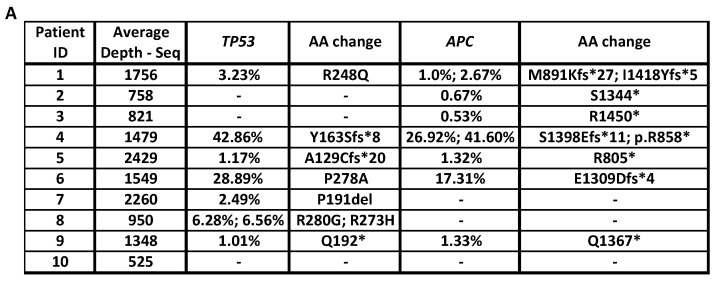

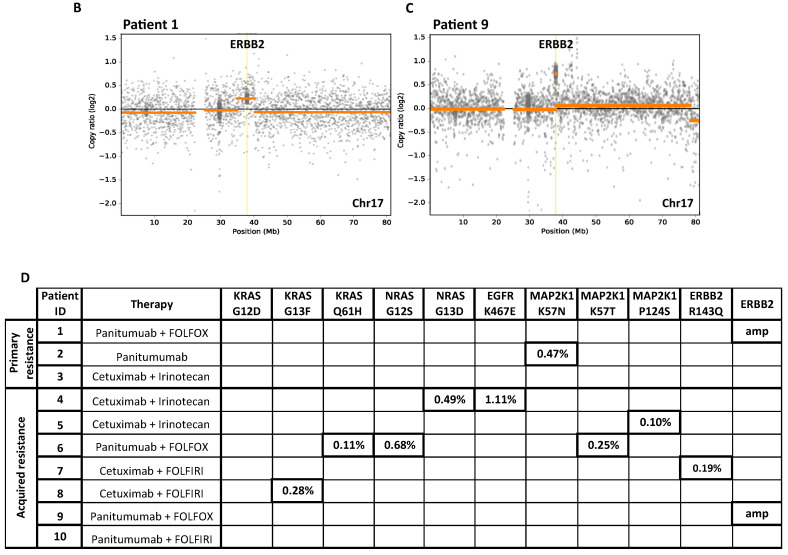
Resistance drivers identified by ctDNA-Seq in mCRC patients at PD to anti-EGFR-Abs. (**A**) Non-silent mutations in the CRC driver genes *TP53* or *APC* identified in the ctDNA and average read depth in ctDNA-Seq. The Variant Allele Frequencies for each mutation are shown. (**B**) Chromosome 17 copy number profile for patient 1 (**C**) and patient 9. (**D**) Drivers mutations/amplifications identified by ctDNA-Seq. Numbers represent the Variant Allele Frequencies of detected mutations.

Up to 25 ng of the ctDNA were sequenced with our error-corrected ctDNA-Seq panel (40 cancer genes, 221kb target region), which includes commonly mutated CRC driver genes (*APC, TP53, FBXW7, PIK3CA, and SMAD2/4)* and known EGFR-Ab resistance driver genes (*KRAS*, *NRAS*, *EGFR*, *BRAF*, *MAP2K1*, *MET, NF1, FGFR2,* and *ERBB2*) [1,2,3,4,5,6,7,8,9,10]. The average read depth in the analysable target region after MBC deduplication was 1388x (Figure 1A). Mutations in the CRC driver genes *TP53* or *APC* were identified in the ctDNA of 9 out of 10 patients (Figure 1A). Genome-wide DNA copy number profiles were reconstructed for all cases to identify gene amplification (Figure S1).

#### 2.1.1. Identification of Drivers of Primary Resistance by ctDNA Sequencing

We next identified likely drivers of resistance to EGFR-Ab in the three patients with primary resistant mCRCs. *ERBB2* amplification was detected in the copy number profile of patient 1 (Figure 1B). *ERBB2* amplifications have previously been shown to confer primary EGFR-Ab resistance [13]. Furthermore, a mutation in the tumour-suppressor gene *NF1* (encoding F1247L) was called in this sample, but this has not been seen in the Cosmic cancer mutation database, and it was not an inactivating mutation (Table S2). No further resistance driver mutations were detected in this patient. In addition, we identified a MAP2K1/MEK1 K57N mutation in patient 2 (Figure 1D). K57N is known to constitutively activate MEK1 in colorectal cancer cell lines [14], and we and others previously showed a role in EGFR-Ab resistance [9,13]. No resistance mechanism was identified in patient 3. Thus, ctDNA-Seq identified an explanation for primary resistance in 2/3 cases (67%).

#### 2.1.2. Identification of Drivers of Acquired Resistance by ctDNA Sequencing

**Figure 2:**
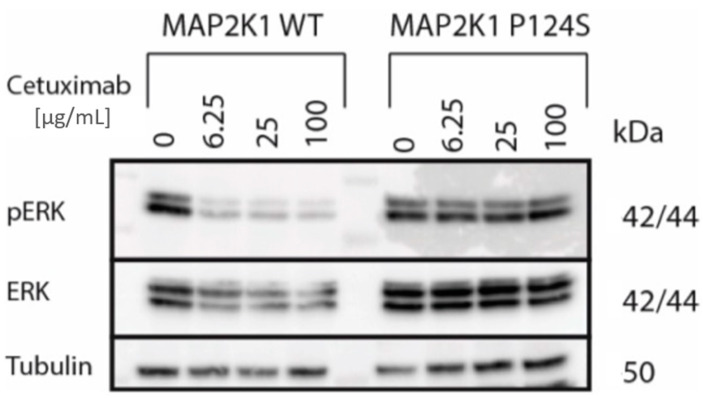
Western blot analysis of parental, MAP2K1/MEK1 wild-type transduced, and MAP2K1/MEK1 P124S transduced DiFi cell line treated with cetuximab for 2 h. Original blot images are provided in Figure S2.

We analysed ctDNA-Seq results from the seven patients with acquired EGFR-Ab resistance. Genetic aberrations that were likely responsible for acquired resistance were detected in 6/7 patients (Figure 1D). Two patients harboured more than one aberration. NRAS G13D and EGFR K467E mutations were found in patient 4, in addition to an NF1 A2511V mutation reported in ClinVar as likely benign [15] (Table S2). NRAS G12S, KRAS Q61H, and MAP2K1/MEK1 K57T mutations were detected in patient 6. A KRAS G13F mutation was identified in patient 8 and a MAP2K1/MEK1 P124S mutation in patient 5 (Figure 1D). P124S is located in the MEK1 protein kinase domain and has previously been shown to confer resistance to BRAF- and MEK-inhibitor therapy in melanoma [16], but its role in EGFR-Ab resistance in CRC was unknown. Expression of MAP2K1/MEK1 P124S and wild-type MAP2K1/MEK1 in the cetuximab sensitive CRC cell line DiFi showed that the mutation rescued ERK phosphorylation and confirmed it as a new driver of acquired cetuximab resistance (Figure 2).

Amplification of *ERBB2* was identified in patient 9 (Figure 1C) and an ERBB2 R143Q mutation (0.19%) in patient 7 (Figure 1D). The latter has previously been described in bladder cancer cell lines as a potential activating mutation, which sensitises to the pan-EGFR inhibitor lapatinib in-vitro [17]. CtDNA-Seq identified no driver of acquired resistance in patient 10 (Figure 1D). This is likely due to low tumour content in the ctDNA as indicated by the absence of clear DNA copy number aberrations in this sample (Figure S1) and *APC* or *TP53* mutations, which had been detected in all other samples.

Together, likely drivers of acquired EGFR-Ab resistance were detected in 86% (6/7) of patients using ctDNA-Seq (Figure 1D). Consistent with prior studies that showed that acquired resistance is often polyclonal [8,9], more than one resistance driver was detected in 2/7 (29%) patients. 

#### 2.1.3. Clonality of Drivers of Primary and Acquired Resistance 

We recently showed that most cancer cells did not harbor any resistance mutations at the time CRCs acquired resistance and progressed on single-agent cetuximab [9]. Whether a similar genetic resistance-gap occurs at acquired resistance in mCRCs treated with a combination of chemotherapy and EGFR-Ab is unknown. Using our established method [9], we assessed the clonality of resistance driver mutations by first correcting VAFs for the influence copy-number states and by subsequently calculating the proportion of cancer cells that harboured resistance driver mutations by comparing against *TP53* or *APC* mutations, which are likely clonal (Table S3). This also corrects for variable tumour contents in different ctDNA samples. Clonality assessment was not possible for patient 9 where an amplification had been detected, as the absolute number of amplified DNA copies in such subclones cannot be assessed; also for patient 10 where no resistance drivers were identified. 

**Figure 3:**
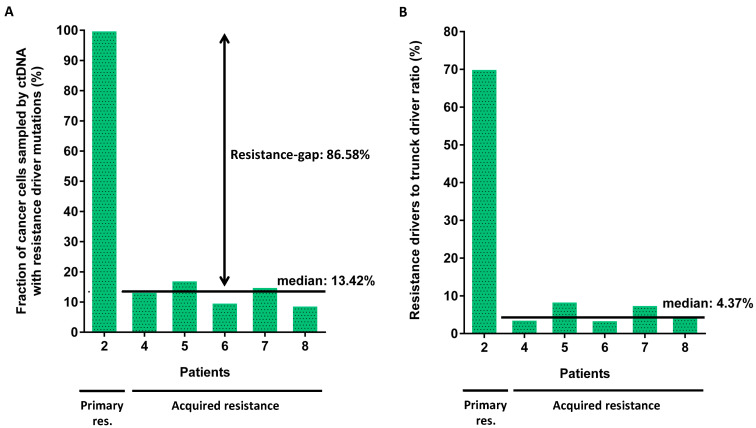
Clonality analysis of EGFR-Ab resistance driver mutations in ctDNA by comparison to truncal CRC driver mutations in *TP53* or *APC*. (**A**) The fraction of cancer cells sampled by ctDNA that harboured EGFR-Ab resistance driver mutations when VAFs are corrected for the influence of copy number aberrations. (**B**) Ratio of the VAF of all resistance drivers combined to the VAF of truncal mutations.

The eight driver mutations found in the remaining five tumours with acquired resistance were only present in a median of 7.65% (range 1.14–17.24%) of the cancer cells sampled by ctDNA-Seq. Therefore, they were subclonal (Table S3). When all mutations in each patient were added together, still only a median of 13.42% (range 8.91–17.24%) of all cancer cells represented in the ctDNA were mutated (Figure 3A). In comparison, when we applied the same analysis to patient 2, which showed primary resistance and a *MAP2K1/MEK1* mutation, this was estimated to be present in 100% of the ctDNA and hence clonal (Figure 3A). 

We used a conservative estimate to define the highest likely cancer cell fraction (see methods), but a potential limitation of this analysis is that the copy number states are estimates as allele-specific copy number data cannot be generated from off-target reads, and this can lead to inaccuracies. Therefore, we also applied a published approach to estimate clonality, which uses the ratio of resistance mutation VAFs to the highest VAF of likely truncal drivers without any correction for copy number status [18]. All drivers of acquired resistance combined per case had a median ratio of 4.37% (range 3.60–8.55%) compared to truncal mutations in either *TP53* or *APC* (Figure 3B). Thus, both approaches support the presence of a considerable genetic resistance-gap at acquired resistance to combination EGFR-Ab and chemotherapy. 

## 3. Discussion

We identified *MAP2K1/MEK1* mutations in 3 and *RAS* mutations in 4 of 10 patients. Hence, *MAP2K1/MEK1* mutations were the second most common driver of resistance in this small series that was predominated by tumours with acquired resistance. While MAP2K1/MEK1 codon K57 mutations have previously been associated with EGFR-Ab resistance [9,13,14], we provide the first evidence that P124S mutations contribute to resistance to EGFR-Ab therapy in mCRC. Together, our results highlight the importance of using ctDNA analysis panels that include a broad range of resistance driver genes beyond *RAS* and *BRAF,* such as *MAP2K1* and *ERBB2* [12,19] to stratify patients to EGFR-Abs optimally. The detection of *ERBB2* amplifications and activating *MAPK2K1* through ctDNA-Seq could furthermore stratify these patients for treatment with trastuzumab or treatment with MEK-inhibitors in clinical trials [20]. The ability of ctDNA-Seq to assess mutation clonality may help select tumours with clonal drivers to avoid targeting subclonal drivers that will likely be futile [21]. 

Importantly, subclonality analyses demonstrated that mutations driving acquired resistance to EGFR-Ab in combination with chemotherapy were confined to small subclones. No genetic resistance drivers were detected in a median of 86.58% of the cancer-derived ctDNA. This defines a genetic resistance-gap in patients with acquired resistance to chemotherapy and EGFR-Abs, similar to the 64% of the cancer cells sampled by ctDNA that had no detectable genetic resistance drivers observed in patients treated with single-agent cetuximab [9]. The clonality estimates are based on published approaches [9,18], but some inaccuracies are possible as these technologies are relatively novel, and not all sources of bias may have been identified. Importantly, our assay’s average sequencing depth was similar to other current ctDNA sequencing technologies [22,23,24], and we have previously shown that the sensitivity of this assay was comparable to other technologies with error correction [12]. Thus, it is unlikely that poor assay sensitivity explains these results. 

Moreover, we previously showed that tumours with a cetuximab-sensitive transcriptomic subtype before single-agent EGFR-Ab treatment changed to a fibroblast- and growth factor-rich subtype at progression and that this stromal remodeling enables non-genetic cetuximab resistance, likely explaining the genetic resistance-gap [9]. Confirming a similar resistance-gap in mCRCs treated with EGFR-Ab and chemotherapy now suggests that non-genetic resistance mechanisms may also be relevant when combination therapy is used. This will require confirmation through studies of tumour biopsies in the future, particularly as several other candidate mechanisms for non-genetic resistance have been described, including myeloid-derived suppressor cells infiltrates [25] or paracrine growth factor secretion by cancer cells [26]. Several of the identified non-genetic resistance mechanisms depend on secreted growth factors and may be clinically targetable through blocking agents. Dissection of these mechanisms may therefore inform rational combination treatments with EGFR-Abs and chemotherapy. Minimally invasive technologies to assess the cancer microenvironment compositions or growth factor secretion in the microenvironment are an unmet need. Developing these could accelerate the interrogation of such understudied resistance mechanisms. 

An alternative explanation for this resistance-gap could be that EGFR-Ab resistance is the consequence of genetic drivers scattered across many genes that are rarely mutated individually and therefore remained unidentified to date. However, our previous finding that cancer-associated fibroblasts increased in PD biopsies without detectable genetic resistance drivers and that these can mechanistically rescue cancer cell growth supported by the non-genetic resistance model [9].

## 4. Materials and Methods

### 4.1. Patients

Ten patients with *RAS* wild-type status mCRCs who received treatment with EGFR-Ab (cetuximab/panitumumab) containing therapy were included in this study. The study has been approved by the Hospital Sírio Libanês Ethics Committee (Study # HSL 2015-22), and all patients provided written informed consent before study inclusion. Information from clinical *RAS* mutation tests of tumour tissue from each patient was available for this study. 

### 4.2. Plasma Samples

Blood samples (15 mL) were collected in EDTA-tubes at the time of clinical progression to EGFR-Ab. Plasma was separated by centrifugation at 800× *g* for 10 min at 4 °C within 2 h after collection. Plasma was spun again at 11,000× *g* for 10 min at 4 °C and stored at −80 °C. ctDNA was isolated using the QIAamp MinElute Virus Vacuum Kit (Qiagen, Hilden, Germany). 

### 4.3. ctDNA-Sequencing

Between 17.6 ng and 25 ng of ctDNA was sequenced per sample using SureSelect^XT-HS^ library preparation (Agilent Technologies, Santa Clara, CA, USA) and custom target enrichment of 40 genes as described [9,12]. Library pools were clustered using a cBot (Illumina, San Diego, CA, USA) and sequenced with 75 bp paired-end reads on a HiSeq2500 (Illumina, San Diego, CA, USA) in rapid-output mode.

### 4.4. Variant Calling

SureCall (version 4.0.1.46, Agilent Technologies, Santa Clara, CA, USA) was used to trim and align fastq reads to the hg19 reference genome with default parameters and to deduplicate using Molecular Barcode (MBC) error correction, permitting one base mismatch within each MBC. Consensus families comprising single reads were removed. The SureCall software was used to determine the average on-target read depth (the average number of reads at each position of the analyzable target regions) and variant calls using the SNPPET function. The DuplexCaller [12] was used to identify mutations supported by duplex reads in the common CRC driver genes *TP53*, *APC*, *SMAD2/4*, *FBXW7*, *PIK3CA*, and in the known resistance driver genes *KRAS*, *NRAS*, *BRAF*, *MAP2K1*, *EGFR*, *FGFR2*, *ERBB2*, *NF1*. Mutations supported by reads with this duplex configuration were inferred to come from double-stranded DNA molecules.

### 4.5. Genome-Wide DNA Copy Number Analysis

BAM files from MBC-deduplication before removing single-read consensus families were used to generate genome-wide DNA copy number profiles with CNVkit [27] (v0.8.1). CNVkit was run in non-batch mode with antitarget average size set to 30 kb. Data from healthy donor samples were used as the normal reference pooled dataset [12]. We then assessed each profile for amplifications of the known resistance driver genes *ERBB2*, *MET*, *FGFR2*, and *NRAS/KRAS*.

### 4.6. Mutation Clonality Analysis

Absolute copy number data was estimated from the genome-wide copy number profiles using the following assumptions: the lowest arm level loss corresponds to copy number 1, the modal chromosome number has a copy number between 2 and 4, and copy number states are approximately equally spaced. For the most conservative clonality estimate, we assumed that only one copy of resistance driver genes is mutated and that all copies of the tumour suppressor genes *TP53* and *APC* harbour the detected mutations as this leads to the highest clonality estimate for resistance drivers. The fraction of cancer cells sampled by ctDNA that harboured a resistance driver mutation at PD was calculated by first correcting VAFs for the influence of copy-number states and then by dividing the corrected VAF of resistance drivers by the corrected VAF of clonal TP53/APC mutations. Referencing the resistance driver mutations against clonal mutations corrects for differences in the admixed DNA from normal cells, which varies between patients. The clonality calculations were performed with formulas from Reference [9].

### 4.7. Generation of MAP2K1 Transgenic DiFi Cell Lines and Western Blot Analysis

HEK293T cells were transfected with pHAGE-MAP2K1 and pHAGE-MAP2K1-P124S (plasmids #116757 and #116428, respectively; a gift from Gordon Mills & Kenneth Scott by means of the Addgene non-profit plasmid repository, Watertown, MA, USA) lentiviral constructs in combination with packaging plasmids psPAX and pMD2.G (plasmids #12260 and #12259, respectively; a gift from Didier Trono, Addgene, Watertown, MA, USA) using TransIT-LT1 (Mirus Bio, Madison, WI, USA). DiFi cells were transduced with the resultant viral supernatants in the presence of Polybrene (8 μg/mL). Transduced wild-type MAP2K1 overexpressing cells were selected using 5 μg/mL Puromycin. Mutant MAP2K1 cells (P124S) were selected by fluorescence-activating cell sorting for GFP-high cells on a Sony SH800.

Cells were treated for 4 h with 6.25, 25, or 100 µg/mL cetuximab or with vehicle control GCTS buffer. Total cell lysates were prepared with NP-40 buffer supplemented with protease and phosphatase inhibitors (Sigma, Merck Group, Darmstadt, Germany). Western blotting used primary antibodies p-ERK (#9101, Cell Signaling Technology, Danvers, MA, USA) and ERK (#9102, Cell Signaling Technology). HRP-conjugated anti-beta Tubulin antibody (#ab21058, Abcam, Cambridge, UK) was used as a loading control. Bands were detected using ECL Prime (GE Healthcare, Chicago, IL, USA) and visualised on a C300 digital imaging system (Azure Biosystems, Dublin, CA, USA). 

## 5. Conclusions

Error corrected ctDNA-sequencing with a targeted panel allows the detection of broad genetic resistance mechanisms in CRCs treated with EGFR-Abs and chemotherapy. This may inform patient stratification to novel therapies and help to avoid ineffective treatment with EGFR-Abs. Furthermore, our data show a genetic resistance-gap after treatment with EGFR-Abs in combination with chemotherapy, indicating a need to investigate resistance mechanisms beyond the well-described genetic point mutations and amplifications in receptor tyrosine kinases and RAS/RAF pathway members.

## Figures and Tables

**Table 1:** Clinical characteristics of metastatic colorectal cancer patients.

Patient ID	Age (Years)	Gender	Histology	Primary Location	Differentiation Grade	EGFR-Ab Therapy	Line of Therapy for Metastatic Disease	Time on EGFR-Ab Therapy	Resistance
1	80	Male	Adenocarcinoma	Right-colon	Moderate	Panitumumab + FOLFOX	2nd	2 weeks	Primary
2	79	Male	Adenocarcinoma	Rectum	Moderate	Panitumumab	3rd	9 weeks	Primary
3	57	Male	Adenocarcinoma	Sigmoid	Well	Cetuximab + Irinotecan (rechallenge with EGFR-Ab)	3rd	10 weeks	Primary
4	58	Female	Adenocarcinoma	Rectum	Well	Cetuximab + Irinotecan (rechallenge with EGFR-Ab)	3rd	16 weeks	Acquired
5	52	Male	Adenocarcinoma	Sigmoid	Moderate	Cetuximab + Irinotecan	2nd	20 weeks	Acquired
6	64	Male	Adenocarcinoma	Rectum	Moderate	Panitumumab + FOLFOX	1st	12 weeks	Acquired
7	41	Female	Adenocarcinoma	Sigmoid	Poor	Cetuximab + FOLFIRI	1st	27 weeks	Acquired
8	53	Female	Adenocarcinoma	Right-colon	Poor	Cetuximab + FOLFIRI	2nd	27 weeks	Acquired
9	46	Female	Adenocarcinoma	Sigmoid	Moderate	Panitumumab + FOLFOX	2nd	29 weeks	Acquired
10	30	Male	Adenocarcinoma	Rectum	Moderate	Panitumumab + FOLFIRI	5th	26 weeks	Acquired

## Supplementary Materials

The following are available online at https://www.mdpi.com/2072-6694/12/12/3736/s1, Figure S1: Log copy ratio plots for 10 patients in our cohort from ctDNA-Seq assay, Figure S2: Original Western blot images relating to Figure 2, Table S1: Molecular analysis performed with tissue biopsy at diagnosis of mCRC patients, Table S2: Somatic mutations called by ctDNA-Seq, Table S3: Fraction of cancer cells sampled by ctDNA that harbored a resistance driver mutation at PD.
