# Supplementary material for: Circulating Tumour DNA Sequencing Identifies a Genetic Resistance-Gap in Colorectal Cancers with Acquired Resistance to EGFR-Antibodies and Chemotherapy
Source: Cancers (Basel). 2020 Dec 11;12(12):3736. doi: 10.3390/cancers12123736 (PMC7764102; doi:10.3390/cancers12123736)

Supplementary Material

Table S1. Molecular analysis performed with tissue biopsy at diagnosis of mCRC patients.

Patient ID	Tissue Biopsy	Molecular test	KRAS				NRAS				BRAF	
			Codon 12/13	Codon 59	Codon 61	Codon 117/146	Codon 12/13	Codon 59	Codon 61	Codon 117/146	Exon 11	Exon 15
1	Primary	Sanger										
2	Primary and lung metastasis	Sanger										
3	Primary	Sanger										
4	Lymph Node	TruSight Tumor										
5	Primary	RT-PCR Taqman Mutation										
6	Primary	TruSight Tumor										
7	Primary	Foundation One										
8	Primary	Foundation One										
9	Primary	Sanger										
10	Primary	Sanger										

Molecular analysis performed

Molecular analysis not performed

Table S2. Somatic mutations called by ctDNA-Seq

Patient ID	chr position	Gene	Protein change	Ref. Allele	Alt. Allele	VAF	Primary Effect	Described in COSMIC	ClinVar clinical significance	Comments	Classification
1	10:123278289	FGFR2	p.E243K	C	T	0.25%	NON_SYNONYMOUS_CODING	No	Unclassified	Extracellular domain mutation	Unknown
	18:45394812	SMAD2	p.L149F	T	A	0.32%	NON_SYNONYMOUS_CODING	No	Unclassified		Unknown
	17:29562661	NF1	p.F1247L	T	G	1.60%	NON_SYNONYMOUS_CODING	No	Unclassified		Unknown
	5:112173956	APC	p.M891Kfs*27	A	AAAGTC	1.00%	FRAME_SHIFT INSERTION	No	Unclassified		Driver
	5:112175540	APC	p.I1418Yfs*5	A	AT	2.67%	FRAME_SHIFT INSERTION	No	Unclassified		Driver
	17:7577538	TP53	p.R248Q	C	T	3.23%	NON_SYNONYMOUS_CODING	Yes	Pathogenic		Driver
2	15:66727455	MAP2K1	p.K57N	G	T	0.47%	NON_SYNONYMOUS_CODING	Yes	Pathogenic		Driver
	5:112175322	APC	p.S1344*	C	A	0.67%	STOP_GAINED	Yes	Pathogenic		Driver
3	5:112175639	APC	p.R1450*	C	T	0.53%	STOP_GAINED	Yes	Pathogenic		Driver
4	1:115258744	NRAS	p.G13D	C	T	0.49%	NON_SYNONYMOUS_CODING	Yes	Pathogenic		Driver
	7:55227932	EGFR	p.K467E	A	G	1.11%	NON_SYNONYMOUS_CODING	No	Unclassified	Affects a cetuximab-binding epitope	Driver
	18:48604790	SMAD4	p.E538*	G	T	2.34%	STOP_GAINED	Yes	Uncertain significance		Driver
	17:29679412	NF1	p.A2511V	C	T	5.23%	NON_SYNONYMOUS_CODING	No	Benign		Benign
	17:7578442	TP53	p.Y163Sfs*8	T	TAG	42.86%	FRAME_SHIFT INSERTION	No	Unclassified		Driver
	5:112175480	APC	p.S1398Efs*11	G	GA	26.92%	FRAME_SHIFT	No	Unclassified		Driver
	5:112173917	APC	p.R876*	C	T	41.60%	STOP_GAINED	Yes	Pathogenic		Driver
5	15:66729162	MAP2K1	p.P124S	C	T	0.10%	NON_SYNONYMOUS_CODING	Yes	Pathogenic		Driver
	10:123239371	FGFR2	p.*705W	T	C	0.12%	STOP_LOST	No	Unclassified	Stop loss with unlikely functional relevance	Unknown
	18:48581159	SMAD4	p.S155G	A	G	0.17%	NON_SYNONYMOUS_CODING	No	Uncertain significance		Unknown
	5:112173704	APC	p.R805*	C	T	1.32%	STOP_GAINED	Yes	Pathogenic		Driver
	17:7578546	TP53	p.A129Cfs*20	A	AG	1.17%	FRAME_SHIFT INSERTION	No	Unclassified		Driver
	18:48575063	SMAD4	p.G86V	G	T	2.26%	NON_SYNONYMOUS_CODING	Yes	Unclassified		Unknown
6	12:25380275	KRAS	p.Q61H	T	G	0.11%	NON_SYNONYMOUS_CODING	Yes	Pathogenic		Driver
	1:115258748	NRAS	p.G12S	C	T	0.68%	NON_SYNONYMOUS_CODING	Yes	Pathogenic		Driver
	15:66727454	MAP2K1	p.K57T	A	C	0.25%	NON_SYNONYMOUS_CODING	Yes	Likely Pathogenic		Driver
	4:153332825	FBXW7	p.Q44R	T	C	0.16%	NON_SYNONYMOUS_CODING	No	Unclassified		Unknown
	5:112175212-216	APC	p.E1309Dfs*4	AAAAG	-	17.31%	FRAME_SHIFT deletion	Yes	Unclassified		Driver
	17:7577106	TP53	p.P278A	G	C	28.89%	NON_SYNONYMOUS_CODING	Yes	Unclassified		Driver
7	17:37864776	ERBB2	p. R143Q	G	A	0.19%	NON_SYNONYMOUS_CODING	Yes	Unclassified	Extracellular domain mutation	Driver
	18:45374881	SMAD2	p.R291Q	C	T	0.12%	NON_SYNONYMOUS_CODING	No	Unclassified		Unknown
	17: 7578275-7578277	TP53	p.P191del	GAG	-	2.49%	FRAME_SHIFT deletion	Yes	Uncertain significance		Driver
8	12:25398281-282	KRAS	p.G13F	C	A	0.28%	NON_SYNONYMOUS_CODING	Yes	Unclassified		Driver
	17:7577100	TP53	p.R280G	T	C	6.28%	NON_SYNONYMOUS_CODING	Yes	Likely Pathogenic		Driver
	17:7577120	TP53	p.R273H	C	T	6.56%	NON_SYNONYMOUS_CODING	Yes	Pathogenic		Driver
9	10:123324058	FGFR2	p.D138N	C	T	0.17%	NON_SYNONYMOUS_CODING	Yes	Unclassified	Extracellular domain mutation	Unknown
	7:55240762	EGFR	p.R669Q	G	A	0.21%	NON_SYNONYMOUS_CODING	Yes	Unclassified	Does not affect known panitumuma-binding epitopes	Unknown
	17:7578275	TP53	p.Q192*	G	A	1.01%	STOP_GAINED	Yes	Unclassified		Driver
	5:112175390	APC	p.Q1367*	C	T	1.33%	STOP_GAINED	Yes	Unclassified		Driver
10	-	-	-	-	-	-	-	-	-		-

Stop or frame shift mutations in tumour suppressor genes and mutations identified as pathogenic or likely pathogenic in ClinVar or described as drivers in the literature were classed as drivers.

Table S3. Fraction of cancer cells sampled by ctDNA that harbored a resistance driver mutation at PD.

Patient ID	Treatment	Resistance	Gene	AA change	Observed VAF	Copies mutated	Copies tumour	Estimated CCF in ctDNA based on TP53/APC	Expected VAF of clonal mutations	Proportion of CCF in ctDNA that harbors a resistance driver mutation*	Clonality	Proprotion of CCF that harbours any resistance driver
2	Panitumumab	Primary	APC	S1344*	0.67%	3	3	0.45%				
			MAP2K1	K57N	0.47%	1	3		0.22%	100.00%	Clonal	100.00%
4	Cetuximab + Irinotecan	Acquired	TP53	Y163Sfs*8	42.86%	3	3	33.34%				
			NRAS	G13D	0.49%	1	3		14.29%	3.43%	Subclonal	13.42%
			EGFR	K467E	1.11%	1	5		11.11%	9.99%	Subclonal	
5	Cetuximab + Irinotecan		TP53	A129Cfs*20	1.17%	2	2	1.17%				
			MAP2K1	P124S	0.10%	1	3		0.58%	17.24%	Subclonal	17.24%
6	Panitumumab + FOLFOX		TP53	P278A	28.89%	3	3	21.31%				
			KRAS	Q61H	0.11%	1	3		9.63%	1.14%	Subclonal	
			NRAS	G12S	0.68%	1	2		10.66%	6.38%	Subclonal	9.87%
			MAP2K1	K57T	0.25%	1	2		10.66%	2.35%	Subclonal	
7	Cetuximab + FOLFIRI		TP53	P191del	2.49%	2	2	2.49%				
			ERBB2	R143Q	0.19%	1	3		1.26%	15.08%	Subclonal	15.08%
8	Cetuximab + FOLFIRI		TP53	R280G	6.28%	2	2	6.28%				
			TP53	R273H	6.55%	2	2					
			KRAS	G13F	0.28%	1	2		3.14%	8.91%	Subclonal	8.91%

*values were capped at 100%

Figure S1

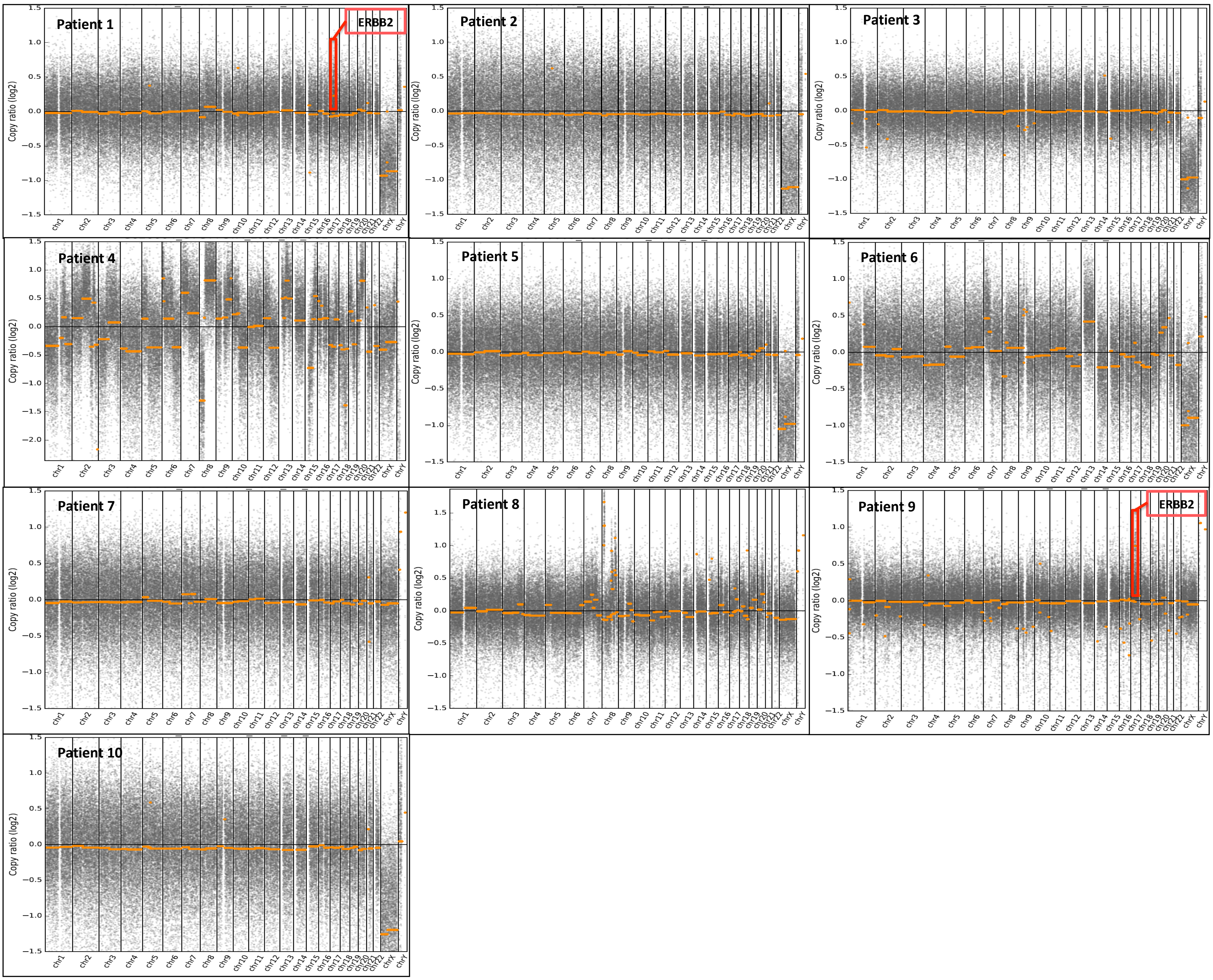

Figure S1. Log copy ratio plots for 10 patients in our cohort from ctDNA-Seq assay.

Figure S2: Original Western blot images relating to figure 2

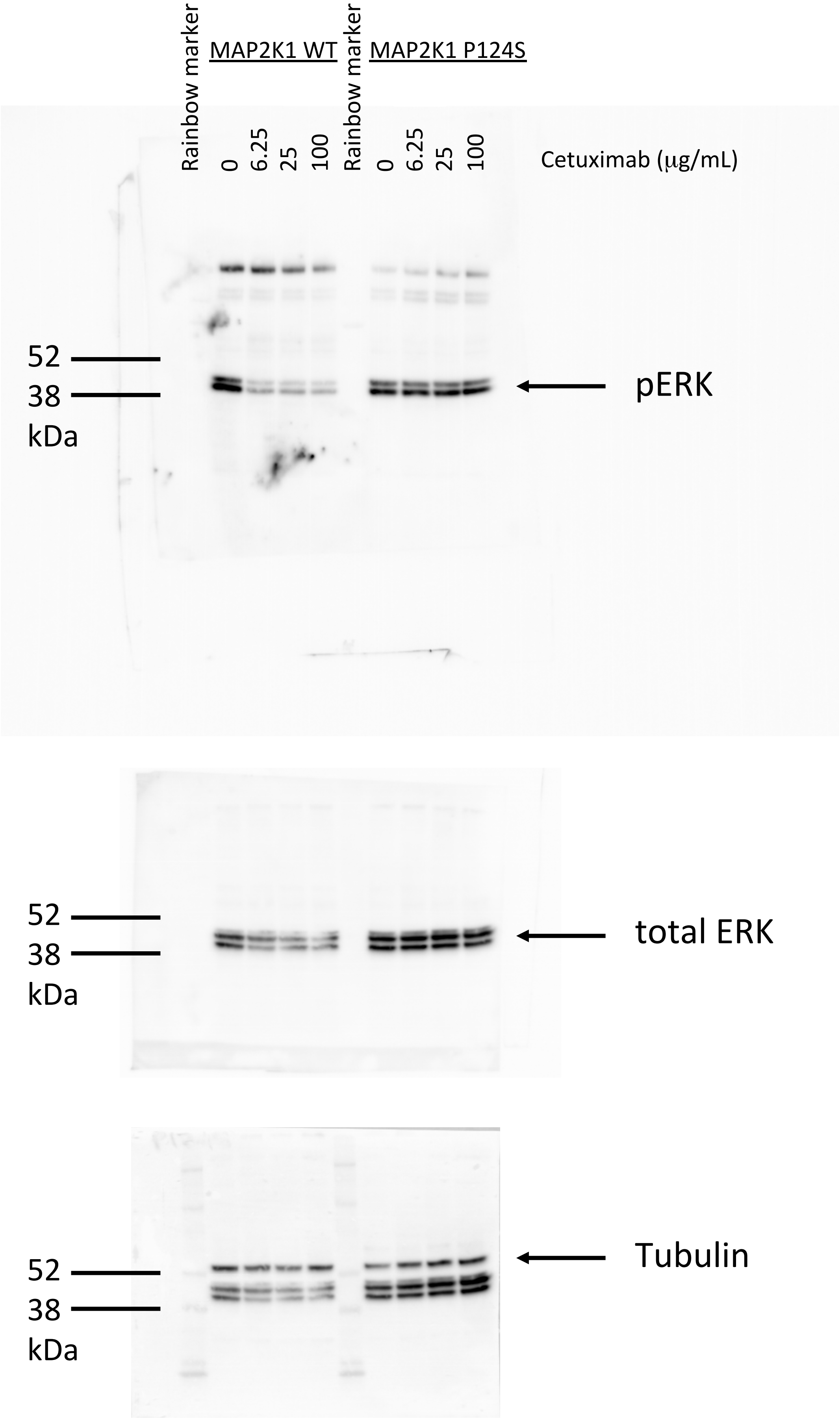

Supplement: Supplementary file 1 [file cancers-12-03736-s001.pdf]
